# Plasmonic tunnel junctions for single-molecule redox chemistry

**DOI:** 10.1038/s41467-017-00819-7

**Published:** 2017-10-20

**Authors:** Bart de Nijs, Felix Benz, Steven J. Barrow, Daniel O. Sigle, Rohit Chikkaraddy, Aniello Palma, Cloudy Carnegie, Marlous Kamp, Ravishankar Sundararaman, Prineha Narang, Oren A. Scherman, Jeremy J. Baumberg

**Affiliations:** 1 NanoPhotonics Centre, Cavendish Laboratory, Department of Physics, JJ Thompson Ave, University of Cambridge, Cambridge, CB3 0HE UK; 2 Melville Laboratory for Polymer Synthesis, Department of Chemistry, University of Cambridge, Lensfield Road, Cambridge, CB2 1EW UK; 3 Department of Materials Science and Engineering, Rensselaer Polytechnic Institute, Troy,, 12180 NY USA; 4 John A. Paulson School of Engineering and Applied Sciences, Faculty of Arts and Sciences, Harvard University, Cambridge,, 02138 MA USA

## Abstract

Nanoparticles attached just above a flat metallic surface can trap optical fields in the nanoscale gap. This enables local spectroscopy of a few molecules within each coupled plasmonic hotspot, with near thousand-fold enhancement of the incident fields. As a result of non-radiative relaxation pathways, the plasmons in such sub-nanometre cavities generate hot charge carriers, which can catalyse chemical reactions or induce redox processes in molecules located within the plasmonic hotspots. Here, surface-enhanced Raman spectroscopy allows us to track these hot-electron-induced chemical reduction processes in a series of different aromatic molecules. We demonstrate that by increasing the tunnelling barrier height and the dephasing strength, a transition from coherent to hopping electron transport occurs, enabling observation of redox processes in real time at the single-molecule level.

## Abstract (web summary)

Plasmons in sub-nm cavities can enable chemical processes within plasmonic hotspots. Here the authors use surface-enhanced Raman spectroscopy to track hot-electron-induced chemical reduction processes in aromatic molecules, thus enabling observation of redox processes at the single-molecule level.

## Introduction

Noble metal nanostructures support enhanced optical properties due to light-induced collective electron oscillations (plasmons), concentrating incident light at metal-dielectric interfaces. The self-assembly of complex nano-architectures allows for near-field coupling in closely spaced plasmonic components forming highly localised intense plasmonic hotspots. Carefully tuning the distance between metal surfaces can yield nanostructures with plasmonic field enhancements approaching three orders of magnitude, enabling strong surface-enhanced Raman spectroscopy (SERS)^1^. One promising nano-architecture is the nanoparticle-on-mirror (NPoM) geometry where individual metal nanoparticles are placed on a flat metal mirror separated by a thin molecular spacer^2–5^. This system functions similarly to a nanoparticle dimer due to the induced image charges of the nanoparticle within the underlying mirror^2–4, 6^. The resulting highly localised plasmonic hotspot gives field enhancements >600 allowing for single-molecule sensitivities^1, 4, 5, 7–12^. Besides their SERS enhancements, plasmonic structures have gained considerable attention for their ability to generate hot charge carriers as a result of plasmonic non-radiative relaxation pathways, which in turn can be used to induce or aid chemical reactions on or near the surface of a plasmonic structure^13–18^. While hot carrier harvesting into semiconductors is well understood, their ability to perform aqueous-phase chemistry remains unclear.

In recent years, several devices have been proposed to utilise such plasmonic hot charge carrier generation, for photocatalytic devices or as a means of harvesting light^16, 17, 19–21^. Despite demonstrated functionality, fundamental understanding of the hot charge carrier generation and subsequent transfer process to adjacent molecular orbitals is yet to be developed. This is in part due to the difficulty of measuring such processes on the nanoscale since they are fast and hard to detect, resulting in most measurements being performed on bulk scales^16, 17, 22^ or in ultra-high vacuum^23^. Difficulties also arise when combining plasmonic interfaces with semiconductors such as ZnO, TiO_2_ or Ag_2_O, since these are photocatalytic on their own, and for this reason are avoided here.

Further combining SERS or TERS (Tip-Enhanced Raman) with electrochemistry allows redox to be driven by applying a bias to the system, injecting or extracting charge carriers from the analytes. It has already been shown that some spectral changes from such bias-induced redox processes can be observed with single-molecule sensitivity^8–10, 24–26^. Redox processes are also observed for photocatalytically induced chemical reactions on the nanoscale^18^. However, using a single intense plasmonic hotspot for both SERS enhancement and the generation of hot charge carriers allows probing of the resulting hot-electron reduction and oxidation behaviours dynamically, with single-molecule sensitivity, through tracking changes in the SERS spectra of adsorbates over time^5^. Here we focus on the generation and transport of hot charge carriers and show the crucial role of molecular binding to the plasmonic substrate as well as the effect of the interaction strength between the charge carriers and the molecules.

## Results

### Hot charge carrier generation in nanoparticle-on-mirror geometry

**Fig. 1:**
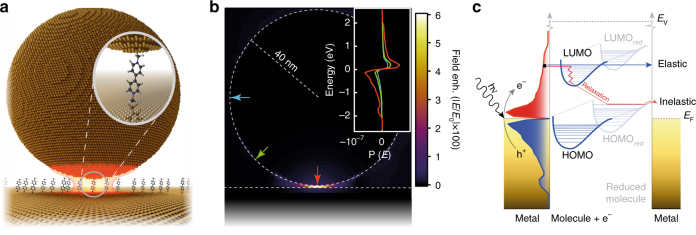
Plasmon generated hot charge carrier transport in a nanoparticle-on-mirror geometry. **a** Self-assembled 80 nm nanoparticle-on-mirror geometry used to elicit field enhancements required for single-molecule spectroscopy. **b** FDTD-calculated electric field enhancement (*E*_max_/*E*_0_ > 600) for structure in **a** at 760 nm. Inset: plasmon-induced hot carrier population for different points around the NPoM geometry (marked with coloured arrows, calculated at 633 nm). **c** Schematic representation which depicts the plasmon-induced hot electron transfer between Au nanoparticles and unoccupied molecular orbitals of adsorbed molecules and the consequent reduction process

The NPoM geometry is used to provide optical field enhancements sufficient for single-molecule sensitivity (Fig. 1a). Separating the gold surfaces by a self-assembled monolayer (SAM) of molecules results in a controllable separation here from 0.9 ± 0.05 nm^22^, to 1.1 ± 0.1 nm^27^, determined by molecular size^28^. The small gap separation results in a tightly confined plasmon coupled mode tuned to yield optimal SERS with the 633 nm excitation laser, giving field enhancements exceeding 600 at 760 nm (Fig. 1b)^2^. The calculated near-field as a function of wavelength shown in Supplementary Note 1 (Supplementary Fig. 1e) indicates the out-going SERS emission is resonant with the NPoM-coupled plasmonic resonance. Atomic-scale morphological features on the gold interfaces further localise the plasmonic hotspot resulting in roughly five-fold higher field enhancements^29, 30^, but while most molecules in the gap contribute very weakly to the final SERS signal, emission is dominated by the individual molecules closest to the centre of these hotspots due to the highly nonlinear Raman enhancement^31^. At nanometre length-scales the molecular spacers act as molecular tunnelling junctions (MTJs), providing low-energy tunnelling pathways for excited charge carriers travelling between the metal surfaces. In contrast to electrochemically driven tunnelling, electron transport here occurs back and forth in both directions with no net charge build-up on either metallic surface around the gap.

The initial spatial distribution of excited hot carriers in the material follows the optical field, while their energy distribution is a property of the plasmonic metal. Calculations using the steady-state linearised Boltzmann equation with ab-initio energy-dependent electron–electron and electron–phonon collision integrals^31, 32^ allow the initial spatial- and energy-resolved carrier distributions at the metal surface to be predicted (Fig. 1b). With increasing distance from the hotspot in the gap, the contribution of the initial high-energy carriers reduces, and that of scattered carriers increases. The carriers that reach the top of the nanoparticle are mostly thermalized. As a result, the spatial region accessible to non-thermal carriers for driving the redox reaction is only slightly larger than the plasmonic hotspot (by ~10 nm, the characteristic mean free path for 1–2 eV carriers). Therefore, most of the hot carriers capable of driving the redox reaction are expected to reach the surface in precisely the region detectable in the experiment, enhancing the efficiency of detection in this region.

### Hot-electron transport behaviour through molecular tunnelling junctions

**Fig. 2:**
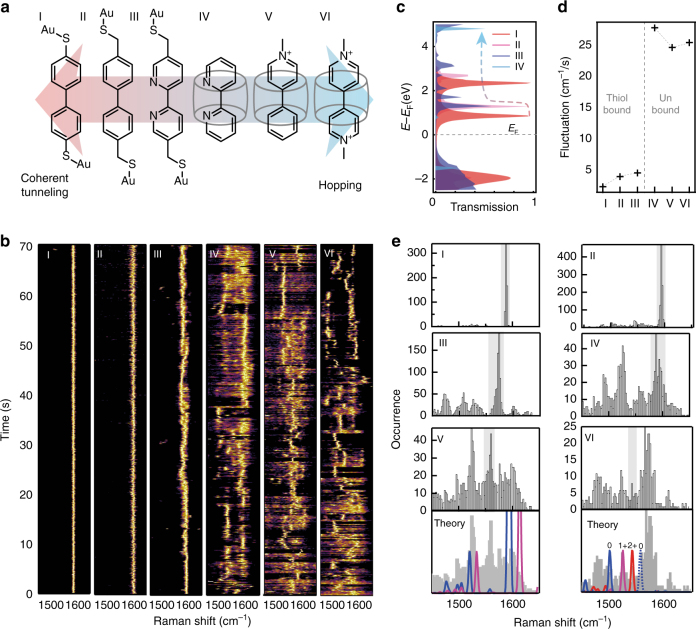
Molecular influence on hot charge carrier transport. **a** Chemical structures of six different molecular tunnelling junctions (MTJ: **I-VI**) used in the hot-electron reduction experiments, sorted by expected tunnelling mechanism. **b** SERS time scans from individual NPoM geometries (normalised), with MTJs **I-VI**, changing the expected electron transport mechanism from coherent tunnelling to hopping (0.1 s integration time taken over 70 s, raw spectra available in Supplementary Fig. 3). **c** Calculated coherent electron transmission spectra for **I-IV**. **d**, Experimentally observed vibrational frequency fluctuations in time for each MTJ. **e** Histograms for each MTJ depicting the occurrence of peaks at each vibrational frequency, grey indicating the unperturbed states, with **V** and **VI** compared to theory (below) where dark grey is exp., *red* depicts the normalised Raman cross-section calculated for the 2 + state (ground state for **VI**), *purple*: 1 + (ground state for **V**) and *blue*: neutral

Typically, at short distances, electron (or hole) transport through MTJs occurs through coherent tunnelling, a fast elastic transport process preventing vibronic coupling that leads to dephasing or relaxation (e.g., oxidation/reduction)^33^. However, if the MTJ develops enough dephasing as a result of molecular length, strong dephasing groups or tunnelling barriers, the dominant electron transfer mechanism changes from coherent tunnelling to hopping^33–38^. This change in transfer mechanism increases the chance of relaxation of the MTJ as a result of temporarily trapping the charge carrier on the molecule (Fig. 1c). To demonstrate this change in electron transport behaviour a series of small and robust molecular spacers with increasing molecular resistance are used as MTJs (Fig. 2a). These molecules have roughly the same length, see Supplementary Note 2, and little or no conformational enantiomers so eliminating contributions to the SERS spectra from conformational changes during the experiments.

A reference molecule, 4,4-biphenyl-dithiol (molecule **I**) is used to show coherent electron transport due to its low molecular resistivity. This arises as a results of the aromatic rings coupling to the thiol groups, which in turn bind to the gold allowing for coherent tunnelling^27, 39, 40^. This conductive link is perturbed by introducing tunnelling barriers in the form of methyl groups between the thiol and phenyl groups (molecule **II**) forming a double-barrier tunnelling junction, increasing the energy at which electrons can tunnel through the junction. This effect can be approximated by calculating the energies of the eigenstates for coherent electron transport, here calculated for molecules (I-VI) placed between two planar gold surfaces (Fig. 2c)^37, 38, 41^. Changing the phenyl rings into pyridyl moieties (molecule **III**) increases the MTJ dephasing strength, resulting in a decrease in coherent transmission probability (Fig. 2c). The effect of the double tunnelling barrier can be drastically increased, while not increasing the distance between the two metal surfaces, by completely eliminating the molecular bond between the Au and the MTJ. To still position the MTJ within the plasmonic hotspot without a linker to the gold, cucurbit[*n*]uril (CB[*n*]) spacers are used^42^, which are macrocyclic molecules where *n* refers to the number of cyclised units. The CB[*n*] sequesters the MTJ and forms the link between the two Au surfaces (molecules **IV**, **V** and **VI**, with this host–guest complex structure depicted in Supplementary Fig. 2).

The MTJs **I-VI** shown in Fig. 2a are ordered by their expected tunnelling mechanism going from coherent tunnelling to hopping as a result of the introduced double barrier and increased dephasing strengths. The additional increase in charge carrier residence time between **V** and **VI** is due to more stable redox states for the latter molecules^43–45^. SERS spectra collected from each of the NPoM-MTJ complexes show pronounced vibrational peaks in the range 1450–1650 cm^−1^. Thus SERS time evolution scans in this range are taken for each of the complexes (Fig. 2b), with peak intensities normalised to highlight the spectral changes. The observed spectral fluctuations are a direct indication that the signals mostly come from a single (or a few) molecules within these nano-constructs^46^.

**Fig. 3:**
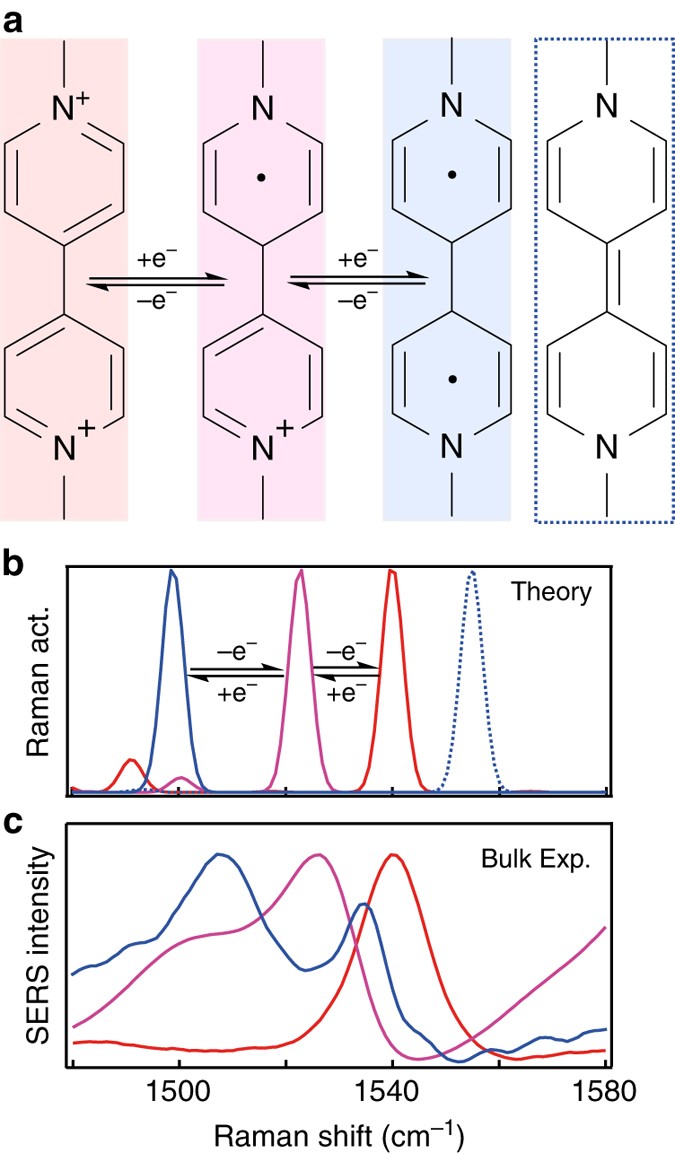
Redox behaviour of molecular tunnelling junction VI (methyl-viologen). **a** Chemical structures of different redox states of **VI**. **b** DFT-calculated Raman spectra and **c** experimental Raman from bulk solutions in which **VI** ^2+^ (*red*) is chemically reduced to **VI** ^1+^ (*purple*) using dithionite, and **VI** ^0^ (triplet, *blue*) using NaBH_4_ or Na_2_S_2_O_4_ in the presence of Au nanoparticles (see Supplementary Fig. 10). The singlet **VI** ^0^ state (*blue dotted line*) is intermittently observed in NPoM experiments

From **I-VI** an increase in fluctuations of the Raman shift of the main peak is observed, with a gradual increase from **I-III** and a dramatic increase for **IV-VI** (Fig. 2b). This shows that elimination of the covalent bond has a drastic effect on the electron transport properties of the MTJ, which agrees with the large increase in energy calculated for the conductive eigenstate between **III** and **IV** (Fig. 2c). At this changeover new transitory peak positions are observed that are stable for short ( < 2 s) periods of time (Fig. 2b). We quantify these fluctuations using the average shift of the main vibrational peak from the average position ${\rm d}\tilde v$ in a given time interval d*t* (1 s), $F = \left| {\frac{{{\rm{d}}\tilde v}}{{{\rm{d}}t}}} \right|$. A steep increase in fluctuations *F* upon elimination of the Au-MTJ bonds is evident (Fig. 2d). This increased fluctuation from **I** through **VI** can also be visualised by plotting the peak occurrence as a function of vibrational frequency (Fig. 2e). A gradual increase in the width of the main occurrence frequency peak in the distribution is observed for MTJs **I-III**, with **IV-VI** showing additional narrow peaks in the histograms representing states that occur more frequently (ground states are highlighted in grey, analysis in Supplementary Fig. 8). The peaks observed for **V** and **VI** are in close agreement with calculated Raman spectra for both ground states and reduced chemical states (Fig. 2e, see Supplementary Note 6 for more details). Shifts corresponding to redox states arise from changes in the conjugated structure as a result of the pyridyl moieties acquiring an extra electron and rearranging the *π*-structure (Fig. 3a).

### Single-molecule redox behaviour

To confirm that the observed vibrational peaks indeed correspond to different redox states, experiments in bulk solution are performed on **VI** using Au nanoparticle aggregates by adding agents known to chemically reduce **VI** ^2+^ (Fig. 3b)^43–45^. In this case the CB[*n*] molecules act to glue together many Au nanoparticles, with SERS integrating over large numbers of nano-gaps in the solution^47^. Adding an excess of sodium dithionite (Na_2_S_2_O_4_) or sodium borohydride (NaBH_4_) reduces **VI** ^2+^ (red trace with peak at 1542 cm^−1^) to **VI** ^0^ (blue trace with peaks at 1505 and 1538 cm^−1^), as confirmed by NMR (Supplementary Fig. 9). Sodium dithionite is used to reduce **VI** ^2+^ to **VI** ^1+^ (purple trace with peak at 1525 cm^−1^), confirmed by NMR (we note reduction using dithionite only forms **VI** ^1+^ in the absence of CB[*n*], with instead the dithionite inducing aggregation of the Au nanoparticles, details in SI). Comparing the SERS from these chemical reductions with the predicted spectra shows a good match in both peak position and trend upon reduction, noting that the best agreement is reached assuming a triplet spin state for **VI** ^0^ in the DFT (Density Functional Theory) calculations (singlet state shown as dotted blue trace in Fig. 3b).

The DFT predictions of the Raman spectra are for isolated molecules (simulations are broadened by 3 cm^−1^ in accordance with the expected broadening at 293 K^48^), in contrast to experiments where they are linked to metal surfaces. This linkage allows the molecular vibrational states to hybridise with the phonons of the metal. However the strongest Raman response remains from the portions localised within the molecule, with decreasing contributions from metal-dominated modes. In addition, contributions from individual molecules are not identical as a result of small configurational differences such as torsion between the pyridyl groups and their proximity to the gold, so an ensemble of molecules results in a Gaussian broadening of the SERS peaks.

**Fig. 4:**
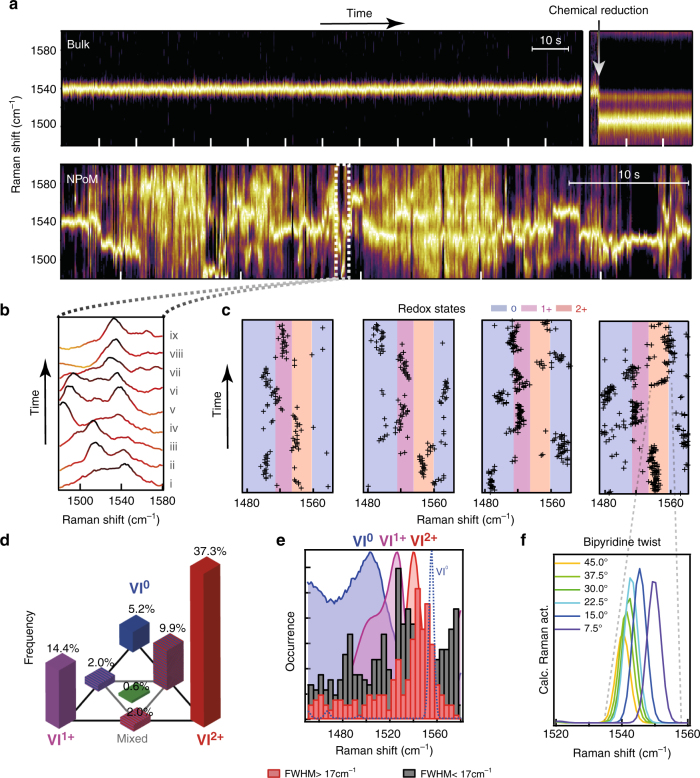
Single-molecule Redox analysis in individual NPoM geometries. **a** Normalised SERS time scans of **VI** with CB[8], ticks represent 10 s: (*top*) bulk measurement in solution with NaBH_4_ added after 115 s (*arrow*); (*bottom*) NPoM measurement taken over 70 s. **b** Short (0.9 s) segment from NPoM time scan showing the reduction and oxidation processes of **VI**. **c** Evolving vibrational peak positions from several NPoM constructs compared to redox states of Fig. 3b. **d** Tri-analyte analysis showing the rarity of seeing more than one redox state, by correlating occurrences of one or several sets of identified SERS peaks (remaining 29.% have no peaks detected, not shown). **e** Distribution of peak positions sorted into histograms of wide FWHM > 17 cm^−1^ (*red*) and narrow peaks FWHM < 17 cm^−1^ (*grey*). **f**, Spectral change in calculated 1542 cm^−1^ peak as a result of twisting between pyridine groups

SERS time scans of CB[*n*]-**VI** ^2+^ multi-gap constructs in solution (bulk) show a stable peak at 1542 cm^−1^ (Fig. 4a). Upon addition of NaBH_4_ (arrow) a nearly instantaneous shift from the **VI** ^2+^ to the **VI** ^0^ spectrum is observed. Comparing the bulk measurements with single NPoM experiments which use the same CB[*n*]-**VI** ^2+^ construct (Fig. 4a, bottom) illustrates the difference in stability of the SERS peak positions. Single-molecule SERS spectra of the NPoM construct show drastic changes in the position of the strongest peak, matching peak positions observed for reduction and oxidation in the bulk experiments (Fig. 3c). Focusing on such a reduction event over 1 s (Fig. 4b), an extra peak is seen appearing ~1525 cm^−1^, corresponding to **VI** ^1+^ (scan ii). This new peak then shifts to ~1490 cm^−1^ (iv-vi) corresponding to **VI** ^0^, and then returns to 1542 cm^−1^ (**VI** ^2+^). These changes in peak position imply that, although part of the Raman spectrum might originate from several **VI** ^2+^ molecules in the plasmonic hotspot, single-molecule events shown by these digital frequency shifts can be readily detected and tracked dynamically. While the Raman cross-sections of **VI** ^1+^ and **VI** ^0^ are larger than **VI** ^2+^, it is clear that additional field localisation must be allowing the selective observation of single molecules in a repeatable fashion^29^. Figure 4c highlights several such reduction events from different NPoM constructs, showing (shaded) the expected vibrational frequencies for **VI** ^2+^ in red, **VI** ^1+^ in purple and **VI** ^**0**^ in blue. Faster time scans with 12 ms integration time show similar dynamics (Supplementary Fig. 4).

Considering the redox states as individual analytes, a bi-analyte type analysis (see ref. ^49^) can be performed by comparing singular and shared events at each time. Fitting peaks within each of the three predominent redox states (1450–1515 cm^−1^, 1515–1525 cm^−1^, 1525–1445 cm^−1^) and comparing singlar and shared occurence events, a clear predominance of singular events is found for each of the states in our tri-analyte analysis (Fig. 4d). Excess events are observed for shared **VI** ^2+^–**VI** ^0^ events, however overall our data show that despite more molecules being present in the hotspot, the redox events are predominantly single-molecule events. If multiple molecules were undergoing redox with similar Raman strengths, the central histogram event (green, simultaneous **VI** ^2+^, **VI** ^1+^, **VI** ^0^) would be dominant, instead of being 10–20 times weaker as shown here. Comparing fitted peaks (Supplementary Fig. 8b), no distinct amplitude differences are observed with Raman shift showing that differences in Raman cross-sections have limited influence on this analysis, with amplitudes predomnantly determined by the molecular positions with respect to the localised field.

Analysing the spectral width ​(Full Width at Half Maximum: FWHM) of the SERS peaks and separating wide from narrow peakwidths, a clear distinction is found (Fig. 4e, full spectral range in Supplementary Fig. 8). The distribution of narrower (FWHM < 17 cm^−1^) and wider (FWHM > 17 cm^−1^) peaks (Fig. 4e), shows the wider peaks centrered around the unreduced **VI** ^2+^. In comparison the narrow SERS peaks are predominanly associated with the **VI** ^1+^ and **VI** ^0^(singlet/triplet) states. This suggests that the majority of **VI** molecules in the gap maintain their 2 + state and contribute weakly to the SERS spectrum, while occassional **VI** molecules at the apex of the plasmonic hotspot undergo these redox processes and dominate the final spectra. This is in line with the findings of the above tri-analyte analysis and supports our conclusion that individual molecule redox behaviour is observed.

Besides the redox processes, additional spectral wandering is also observed within the vibrational frequency range for each redox state. We suggest that this arises from torsion between the two bipyridine rings starting from 45° which is the relaxed geometry for **VI** ^2+^, to planar, which is favoured by **VI** ^1+^ and **VI** ^0^ (Fig. 4f).

The 633 nm laser wavelength used here cannot directly excite interband transitions in Au, CB[*n*], or **VI**. Since gold and CB[*n*] are also unable to induce photo-chemistries directly, then the mechanism of direct plasmonic injection via hot carriers into the LUMO (Lowest Unoccupied Molecular Orbital) levels of **VI** is the only mechanism available. This provides the cleanest evidence yet of hot electron plasmo-chemistry, and thus forms a test-bed for understanding more about the precise mechanisms at the single-molecule level.

**Fig. 5:**
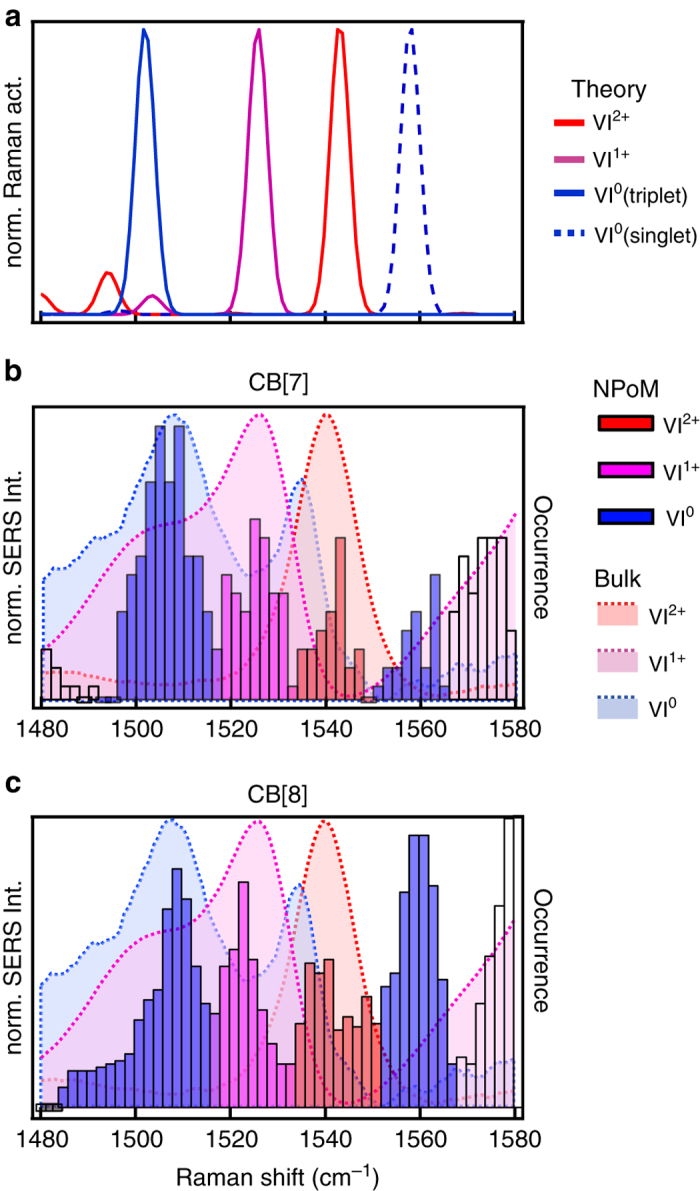
Occurrence of different **VI** redox states in CB[*n*]. **a** DFT calculated peak positions for four different redox states of **VI**: 2 + , 1 + , 0 (singlet), 0 (triplet). **b**, **c** Histograms of single-molecule vibrational peak occurrence from NPoM constructs using **b** CB[7]-**VI**, and **c** CB[8]-**VI** complexes, compared to bulk reduction experiments (shaded areas)

Subtle effects are observed depending on the molecular environment. When comparing NPoM geometries assembled using CB[7] (Fig. 5b) or the more spacious CB[8], which have the same 0.9 nm gap size but different cavity diameters (0.73 nm vs. 0.88 nm, respectively)^42^, the **VI** ^2+^ (1542 cm^−1^), **VI** ^1+^ (1525 cm^−1^) and **VI** ^0^ (1500 cm^−1^) are all observed, but in CB[8] the vibrational state at 1560 cm^−1^ is distinctly more pronounced (Fig. 5c). This latter state closely matches the theoretically predicted spectra for **VI** ^0^ with the more energetically favourable singlet spin state (dotted blue line in Fig. 5a). While this suggests interactions between spin and confinement, different possibilities need to be further explored to ascertain if this striking difference in pairing of the introduced electrons arises as a result of the increased space within the CB[8] cavity, which allows for a more optimal geometry of **VI** ^0^ (e.g., rotational freedom or distance from Au surface). It is also possible there are effects due to the inclusion within the CB[8] cavity of another molecule, or to differences in hydrophobicity between CB[7] and CB[8]. However, it clearly highlights the ability to examine transition redox states of individual molecules dynamically, and the effects of confinement. Additional peaks are observed at 1580 to 1600 cm^−1^, which do not match either the calculations or the solution chemical reduction experiments, and have therefore been left unassigned (white). However they appear to be related to a VI^0^ state as they frequently occur together.

## Discussion

The reliable construction of such plasmonic cavities, which tightly trap the optical field, opens up new generations of photo-chemical investigations tracing out the influence of this plasmonic tunnelling redox. While the potential range of molecules that can be reduced/oxidised using this method is not yet known, the calculated hot-electron distributions (Fig. 1b) suggest reduction potentials up to 1.96 eV (corresponding to the excitation laser at 633 nm). It would thus be interesting to explore the excitation wavelength dependence, requiring experiments with tuneable SERS excitation^50^.

Here, we explored the redox dynamics of single molecules that harness hot electrons produced from the plasmons inside gold. Shifts of vibrational frequencies with charge state allow clear identification of the transient redox state of single molecules, and the spin state of the paired electrons. Slowing the dynamics allows us to see this in ambient conditions, but higher-speed spectroscopy should enable a wide variety of behaviours to be explored, and in particular allow control of the redox states of single molecules with light. We also show the crucial role of binding to the surface and demonstrate that a tunnelling-to-hopping transition can be induced while maintaining the  ~ 1 nm surface-to-surface distance between conducting contacts.

## Methods

### Sample preparation

Atomically smooth Au substrates are prepared by evaporating 100 nm of Au onto silicon wafers at 1 Å/s using an e-beam evaporator, at 1 × 10^−7^ mbar. Then 1 × 1 cm silicon pieces are glued on the Au using epoxy glue (Epo-Tek 377) at 60 °C. After curing the glue for 2 h at 150 °C and slowly cooling to room temperature the silicon pieces are peeled off taking with them the deposited Au film with a surface roughness mirroring that of the silicon wafer^51^.

SAMs of biphenyl-4,4′-dithiol: **I** (Sigma Aldrich, 95.%), 4,4′-bis(mercaptomethyl)biphenyl: **II** (Sigma Aldrich, 97.%) and 5,5′-bis(mercaptomethyl)-2,2′-bipyridine: **III** (SigmaAldrich, 96%) are deposited by submerging the Au surface in a 1 mM solution in ethanol (Sigma Aldrich, absolute, ≥99.5%) for 2 h. The resulting substrate is thoroughly rinsed with ethanol, sonicated for 1 min in ethanol and blown dry using nitrogen. For the CB[*n*] samples the support is submerged for 2 h in a 1 mM solution of 2:1.molar ratio of either bipyridine: **IV** (Sigma Aldrich, >99.%), 1-methyl-4-phenylpyridinium iodide: **V** (Sigma Aldrich, ≥98.%) or methyl viologen dichloride: **VI** (Sigma Aldrich, 95.%) with either CB[7] or CB[8]. The samples are rinsed using de-ionised water and blown dry using nitrogen. Au nanoparticles (obtained from BBI Solutions, 80 nm, citrate capped) are deposited by resting a droplet of the Au suspension on the coated substrates. After 15 s the sample is flushed with de-ionised water and blown dry using nitrogen. For bulk measurements 1 ml of Au solution (60 nm, citrate capped) is combined with 20 µl of a 1 mM CB[7]-**VI** complex [1:1] solution. For reduction experiments using NaBH_4_ the same procedure is repeated with 5 mg of NaBH_4_ added after 115 s to reduce the **VI**. For bulk NaS_2_O_3_ reduction experiments 1 ml of 60 nm Au nanoparticles is combined with 20 µl of a 1 mM solution of **VI** (without CB[*n*]) which results in a similar aggregation process, and 5 mg of NaS_2_O_3_ is added to reduce the **VI**. Adding NaS_2_O_3_ in the presence of CB[*n*] under laser illuminations forms **VI** ^**0**^ instead of **VI** ^1+^, see Supplementary Note 6.

### Experimental setup

Individual NPoM constructs are identified using dark-field microscopy and scanning electron microscopy (Supplementary Note 1). Ensuring the nanoparticles are several microns apart allows spectroscopic probing of individual constructs both in dark-field as well as in SERS. To collect Raman spectra a 632.8 nm HeNe laser is coupled into an Olympus BX51 dark-field microscope. The sample is illuminated with a 100×, NA 0.8 dark-field objective. The laser power on the sample is adjusted to 0.34 mW. The signal is collected through the sample objective, spectrally filtered with a dichroic mirror and long pass (edge) filter. The signal is analysed using an Andor Shamrock spectrograph and Andor Newton EMCCD. Single NPoM geometries are identified using dark-field microscopy and their plasmonic properties are analysed by dark-field scattering spectra, which are recorded through the same objective using a TEC-cooled Ocean Optics QE65Pro spectrometer. Bulk measurements were taken in a 1 ml cuvette using an inVia Raman microscope by Renishaw with a 785 nm laser. Bulk samples are illuminated from the top using a 5×, NA 0.22 objective.

### Simulations

*DFT*. For Raman and density of states simulations, molecular structures are pre-optimised using the Merck Molecular Force Field (MMFF94), with the main optimisation performed by the Gaussian09 package. Vibration spectra are calculated using the hybrid B3LYP/6-31G(p) functional using the CPCM solvation model for water. A scaling factor of 0.975 is used.

*FDTD ​(Finite-Difference Time-Domain)*. Three-dimensional numerical simulations are carried out using Lumerical FDTD Solutions v8.12. The Au NP is modelled as a sphere of diameter 80 nm on top of an infinite dielectric sheet of refractive index 1.45 and thickness of 0.9 nm. Underneath this dielectric sheet, a thick Au layer is placed in order to replicate the experimental NPoM geometry. The dielectric function of Au is taken from Johnson and Christy^52^. The nanoparticle is illuminated with a plane-wave (Total-Field Scattered-Field: TFSF source source) with electric field perpendicular to the substrate, from an angle of incidence of 55°. To obtain efficient numerical convergence, we use perfectly matching layer boundary condition with proper meshing in the gap with 0.2 nm^28^.

Hot carrier generation and transport predictions: We calculate electronic states, phonons, optical and electron–phonon matrix elements, and transform them to a Wannier basis using the DFT software JDFTx. We then calculate the carrier-energy resolved imaginary dielectric function Imє(*ω*,*E*) due to direct and phonon-assisted transitions, and the energy-dependent carrier mean free path *λ*(*E*) and the resulting scattered energy distribution *P*(*E*|*E*’) due to electron–electron and electron–phonon scattering, using Fermi’s golden rule. We then efficiently evaluate the multi-dimensional integrals in ref. ^31^. to obtain the final hot carrier flux by first evaluating the integral at specific directions of $R = r - r'$ using the 1D Green’s function (exp(-*x*/*λ*)) on a tetrahedral mesh, and then integrating over directions of **R** using Monte Carlo sampling with 10^4^ samples.

*Coherent electron transmission spectra*. Planar gold electrodes (consisting of a relaxed periodic array of Au unit cells) with a (111) surface structure are placed on either side of the MTJs using Virtual NanoLab (VNL) and the final geometries were optimised using the Atomistix Toolkit (ATK) implementation of the LDA basis set. Electron transmission spectra were calculated using the extended Hückel basis set, see Supplementary Note 4 for more details^53–56^.

### Data availability

Data used for this article is available at https://doi.org/10.17863/CAM.11881.

## Electronic supplementary material

Supplementary Information
