# Supplementary material for: Plasmonic tunnel junctions for single-molecule redox chemistry
Source: Nat Commun. 2017 Oct 20;8:994. doi: 10.1038/s41467-017-00819-7 (PMC5714966; doi:10.1038/s41467-017-00819-7)
Supplement: Supplementary file 1 — Supplementary Information [file 41467_2017_819_MOESM1_ESM.pdf]

Supplementary Note 1: Dark field measurements and Scattering properties of NPoM geometries

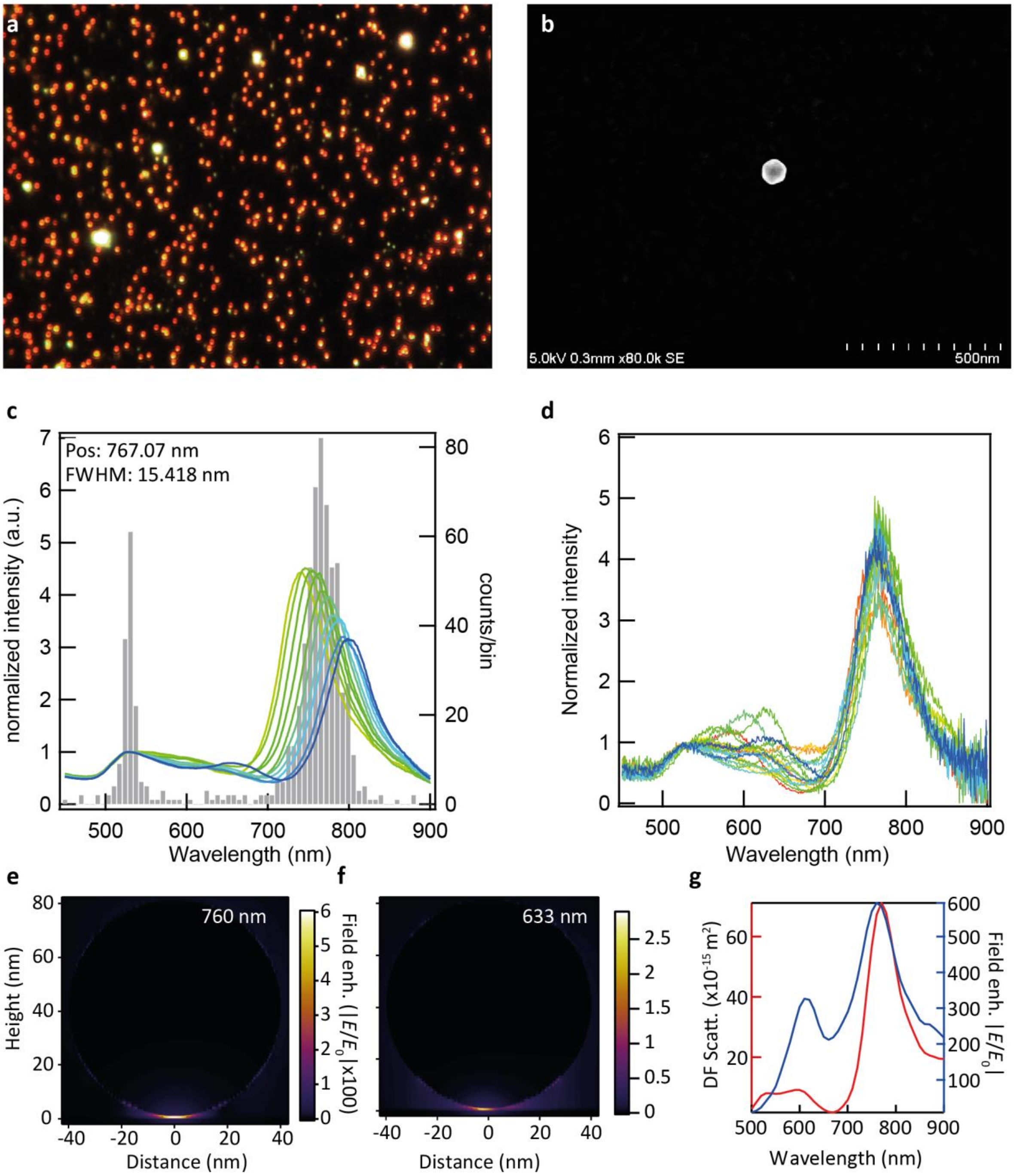

Supplementary Figure 1: Dark field scattering properties of individual nanoparticle on mirror geometries separated by a molecular spacer. **a**, Dark field (DF) scattering image of an 80 nm Au nanoparticle on Au mirror sample with CB-**VI** as a spacer molecule. **b**, Typical SEM image showing the nanoparticle on mirror geometry is formed by single NPs, while NP shapes vary between spherical, icosahedral, and rarer triangular and pentagonal bipyramids. **c,** Dark field scattering spectra sorted by coupled mode position and averaged over 40-80 spectra, showing the variation in coupled mode position throughout a typical sample. **d**, Individual dark field scattering spectra with the same coupled mode position compared, showing the differences in scattering intensity in the shorter wavelength region between 600-700 nm. **e**-**f**, FDTD-calculated electric field enhancement at (e) 760 nm and (f) 633 nm. **g**, FDTD-calculated electrical field enhancement as a function of wavelength.

NPoM geometries form highly reproducible plasmonic hot-spots which can easily be identified using dark-field microscopy (Supplementary Figure 1a), and can be accessed with high numerical aperture (NA) incident light.

Scanning electron microscopy (SEMs) of the samples confirm that the observed NPoM geometries indeed consist of individual 80 nm Au nanoparticles on a flat Au surface. Using a high NA 100x objective and a diffraction limited collection spot, dark field scattering spectra can be collected from individual nanoparticle-on-mirror geometries. Collecting dark-field scattering spectra from ~1000 constructs shows the high degree of reproducibility of this geometry and helps visualize the degree of spectral variation within the sample. In Figure 1c, dark field scattering spectra from NPoM geometries are sorted and averaged on their coupled mode position. Figure 1d shows the spectral variation between individual spectra which all have the same coupled mode wavelength, showing limited spectral variation in the range of 600 nm-700 nm. SERS spectra are collected using the same diffraction limited spot ensuring the collected signal originates from a single plasmonic hot-spot underneath each NPoM geometry.[1] For this geometry and nanoparticle size, the calculated highest field enhancement is found at 760 nm (Supplementary Figure 1e), and at 633 nm gives lower field enhancement for in-coupling of the pump laser (Supplementary Figure 1f). However we obtain the largest SERS signals experimentally for 633 nm because although dark field scattering is weaker at 633 nm, a large near-field enhancement is calculated for this wavelength (Supplementary Figure 1g), which gives the strong SERS.

Supplementary Note 2: Molecular configurations

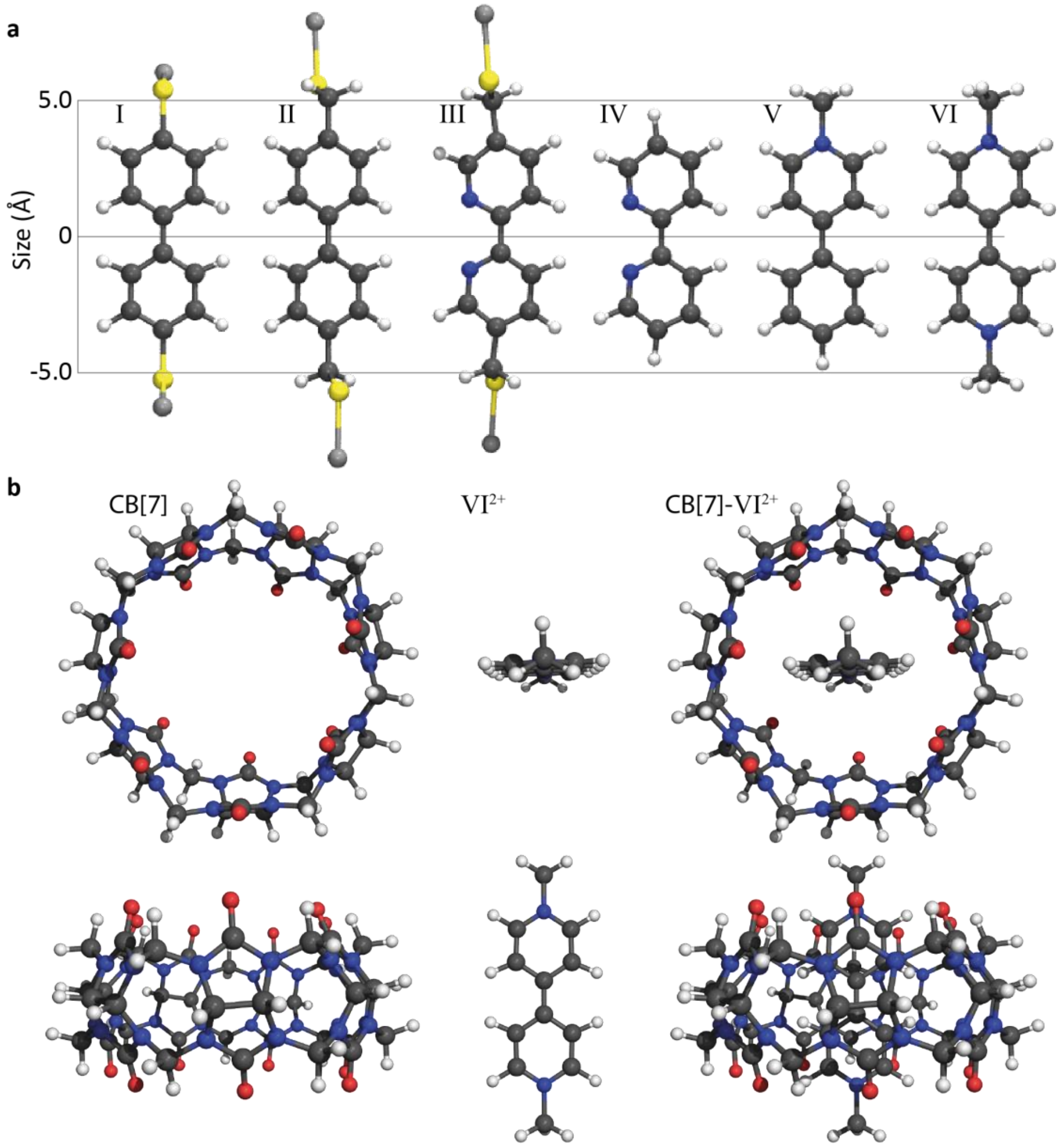

Supplementary Figure 2: Molecular spacer size comparisons. **a**, Absolute lengths of MTJ **I-VI**. Note molecules self-assemble at an angle reducing the metal-metal distance between the two surfaces with respect to the length of the molecule. **b**, Molecular structure of the CB[7]-$\mathbf{VI}^{2+}$ complex, where the CB[7] forms the linker between the Au surfaces and the $\mathbf{VI}^{2+}$ is sequestered in the molecular cavity, positioning it within the plasmonic hot-spot. This molecule **VI** is predicted to sit at an angle in the CB[7], slightly elongating the CB[7] macrocyclic structure.

For molecular tunnelling junctions in the NPoM geometry, the molecules **I** through **VI**, depicted in Supplementary Figure 2a, are used. For molecule **I-III** the molecules are self-assembled using their thiol groups (depicted in yellow) to bind to the Au surface as a robust compact SAM. For MTJs **IV-VI** a CB[*n*] molecule is used to position the MTJ in the plasmonic gap. The hydrophobic cavity of the CB[*n*] sequesters the MTJ (see Figure 2b), whilst the carbonyl portals (depicted in red) bind to the Au surface. In both cases the MTJs are expected to be positioned at an angle with respect to the surface reducing the actual distance between the two metal surfaces. For the latter case we assume the spacing approximates the known separation distance for CB[*n*] spacer molecules, which matches our spectroscopic resonances (Supplementary Figure 1). The CB[*n*] acts as a MTJ on its own but since it has a much lower Raman activity and almost no significant Raman modes in the range 1450-1650 cm^{-1} with respect to **IV-VI** its contribution to the final Raman spectra is minimal (as shown for constructs with CB alone).

Supplementary Note 3: SERS measurements

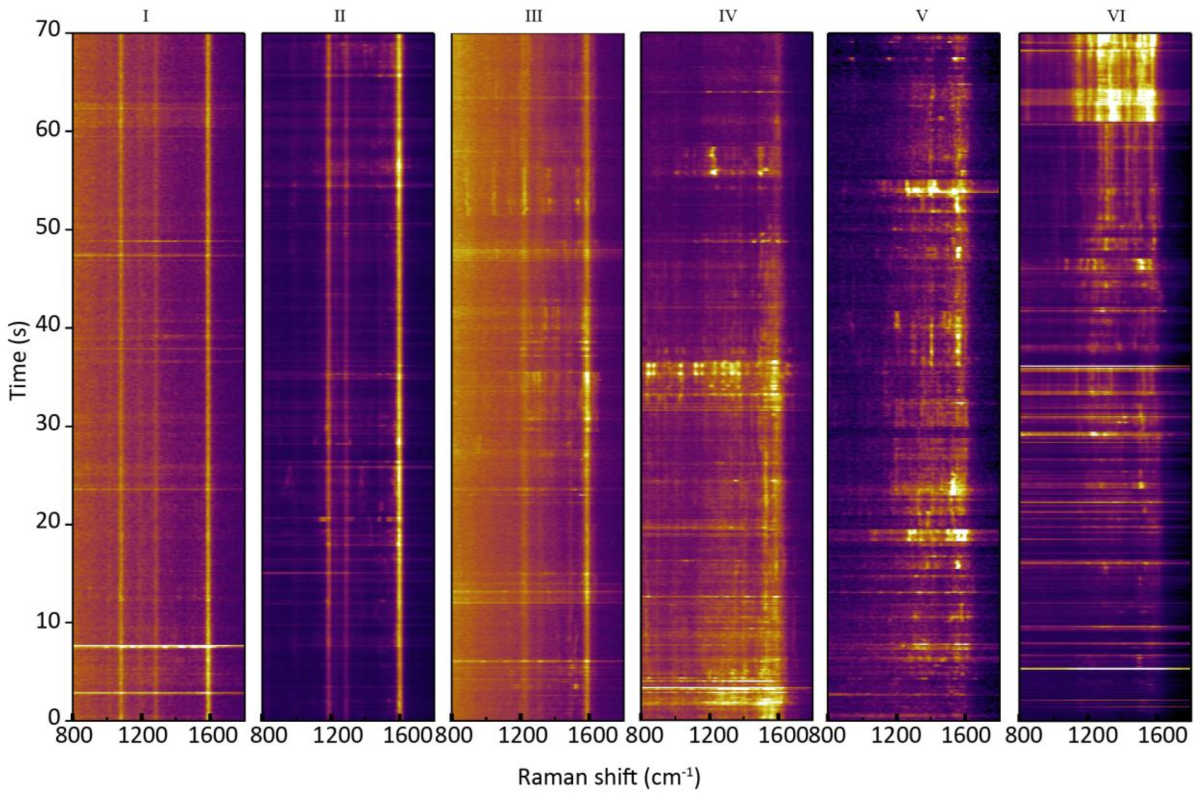

Supplementary Figure 3: SERS time scans from individual NPoM geometries for each respective MTJ over the range 800-1700 cm^{-1}. Scans show most pronounced peaks in the region of 1450 - 1600 cm^{-1} (normalised spectra are shown in Figure 2).

Time scans of the NPoM-MTJ constructs show both the increase in fluctuations of the vibrational frequency as a result of the increased molecular resistivity, as well as also an increase in SERS intensity fluctuations. The exact cause of the intensity fluctuations is not clear but we suspect it is in part a result of the change in redox states that come with drastically different Raman activities. Longer lasting intensity changes, observed for example at the end of the **VI** time scan, we attribute to morphological changes in the Au as observed by Benz *et al*.[2]

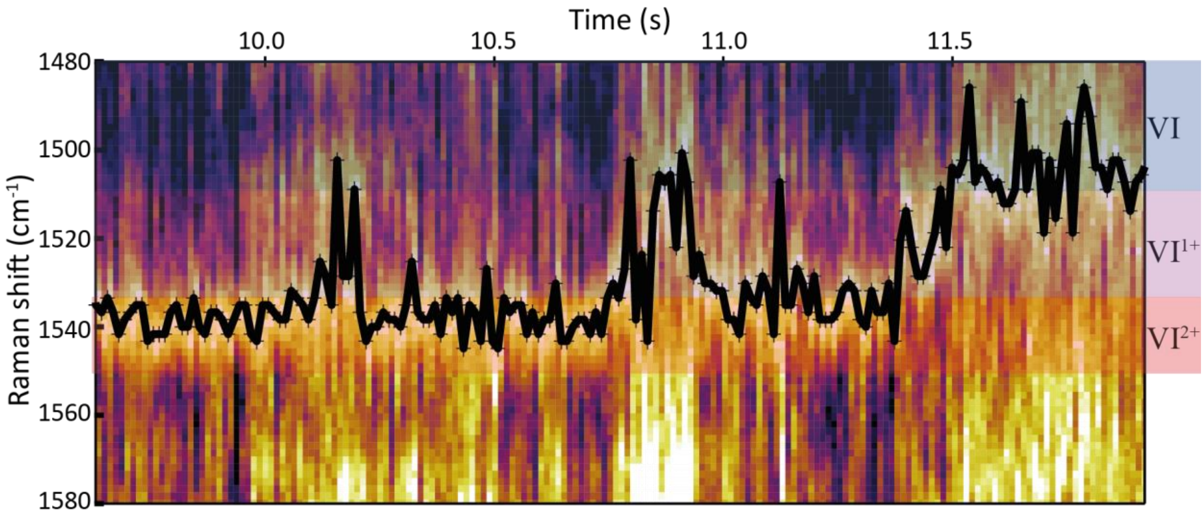

Supplementary Figure 4: SERS time scan of MTJ **VI** taken with 12 ms integration time. High speed SERS spectra show a dominant VI^{2+} state from 9.5 s to 10.7 s, shifting to a dominant VI^{0} state from 11 s onwards (shaded areas indicate redox states based on the bulk reduction experiments).

When increased SERS signal are seen, shortened integration times can be used for fast SERS time scans to gain increased time resolution (Supplementary Figure 4). For this time scan, a 12 ms integration time was used which clearly shows as consistent with the main text, that these reduction and subsequent oxidation processes also occur at faster rates than can be observed using 100 ms integration times. Tentative assignments of the redox states are highlighted in Supplementary Figure 4, though fluctuations in the vibrational frequencies appear which we attribute to torsion of the two bipyridine groups. The effect of the torsion between these groups for VI^{2+} was calculated using DFT calculations (Supplementary Figure 6).

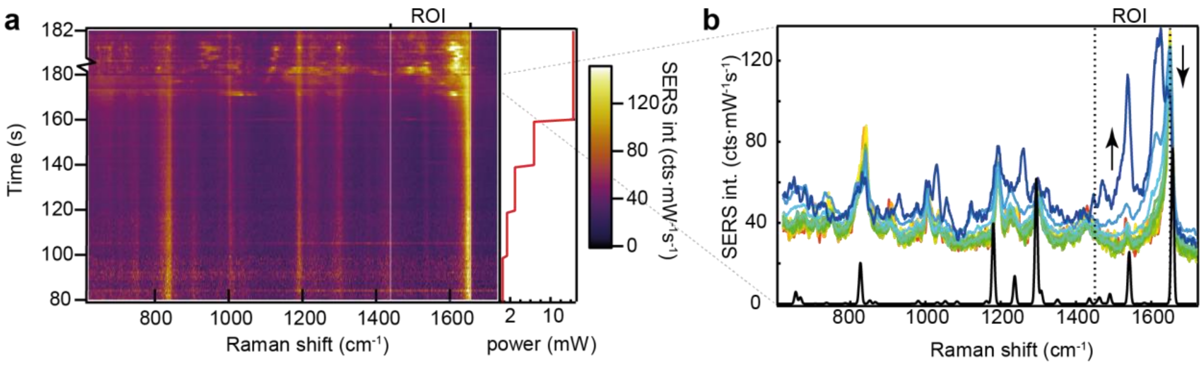

Supplementary Figure 5: SERS characteristics of nanoparticle aggregates as a function of laser power. **a**, SERS time scan for a nanoparticle aggregate containing $\mathbf{VI}^{2+}$ while gradually increasing the laser power from 2 mW to 14 mW. **b**, SERS spectra showing the change in peak ratios as a result of the increased laser power on the nanostructures in (a), showing a distinct increase in the 1545 cm^{-1} mode and a decrease in the 1650 cm^{-1} mode.

Differences in Raman spectra are observed between bulk measurements and NPoM geometries. This is likely a result of changes in the gold nanoparticle morphologies at increased laser powers in the latter case. In bulk solution, the $\mathbf{VI}^{2+}$ spectra can be matched (including peak ratios) to DFT calculations. However, in the NPoM experiments peaks around 1545 cm^{-1} are more pronounced than the normally (bulk) dominant peak at 1650 cm^{-1}. This we think is a result of the formation of 'pico-cavities' in the junctions between the gold nanoparticles, increasing the SERS intensities and changing the Raman selection rules resulting in different

intensity ratios in the spectra.[2] By contrast, the laser power per junction in the bulk experiments is not enough to deform the gold. Exposing a single CB[*n*]-AuNP aggregate to laser light shows this transition from the bulk spectra to the NPoM spectra (Supplementary Figure 5). By gradually increasing the laser power from 2 – 10 mW (through a 50x 0.8 NA objective) and taking Raman spectra with 1 s integration times, changes in the SERS spectra are observed after a laser power of 14 mW is exceeded (Supplementary Figure 5a). After this transition the SERS intensity is increased, allowing for collection at 0.1 s integration times (180-182 s in Supplementary Figure 5a). Change in the Raman spectra occur as a result of the morphology changes in the gold nanoparticles (Supplementary Figure 5b), with the most pronounced change being an increase in the 1545 cm^{-1} mode and a decrease in the 1650 cm^{-1} mode.

Supplementary Note 4: DFT Calculations

Predicted Raman activity spectra were calculated using the Gaussian implementation of the Becke three-parameter Lee-Yang-Parr (B3LYP) hybrid exchange-correlation functional, using the 6-31G(d) basis set. The CPCM solvation model was used to model the surrounding water phase, assuming that in the dry NPoM system, water is still present in the nanometre sized cavities in and around the CB[*n*] spacers.

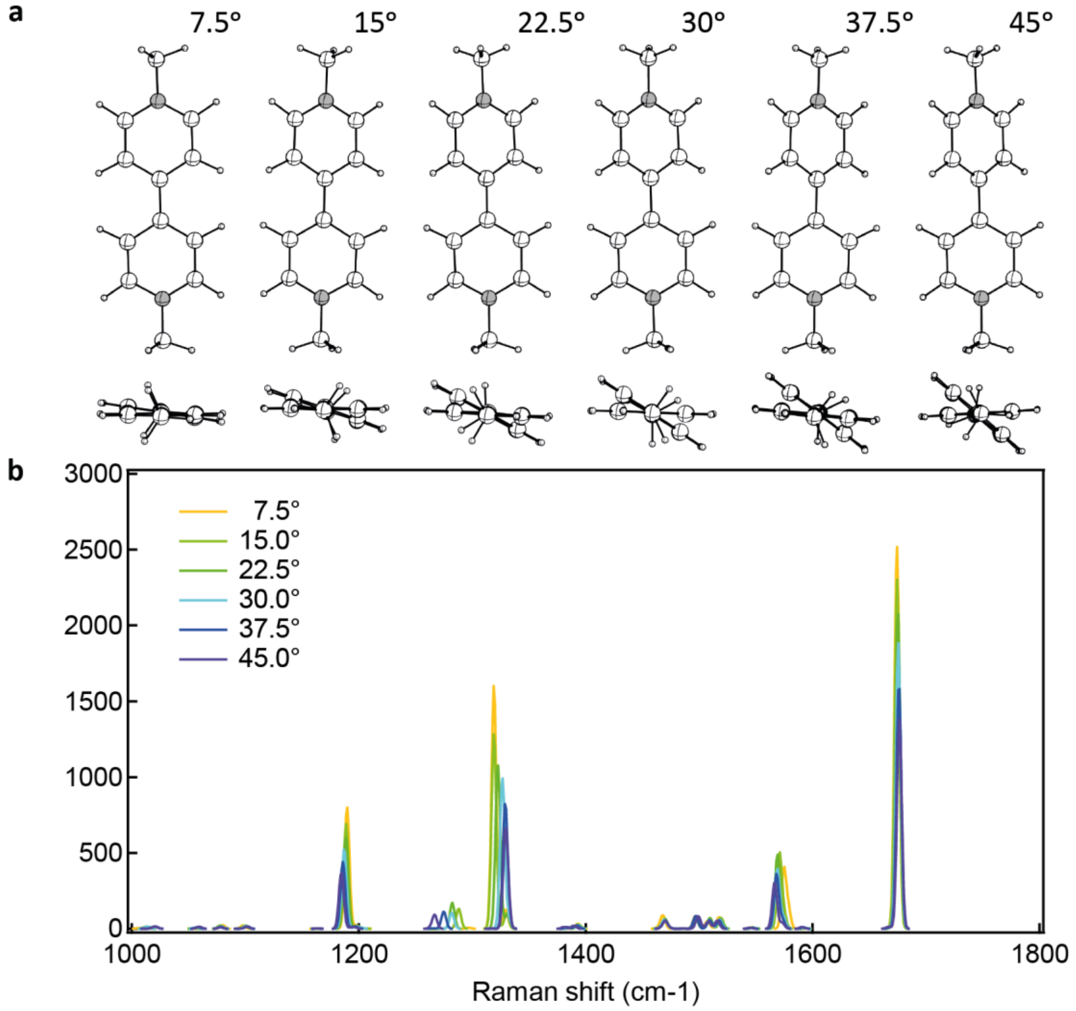

Supplementary Figure 6: Calculated Raman activity showing the effect of torsion angle between the two pyridine groups on the resulting $\mathbf{VI}^{2+}$ spectra.

By fixing dihedral angles around the central (6,7) atoms the twist between the two pyridine rings can be locked preventing the structure reaching a fully energy minimized geometry during optimisation. In this way the effect of torsion between the two pyridine groups on the Raman activity can be visualized (Supplementary Figure 6).

a

b

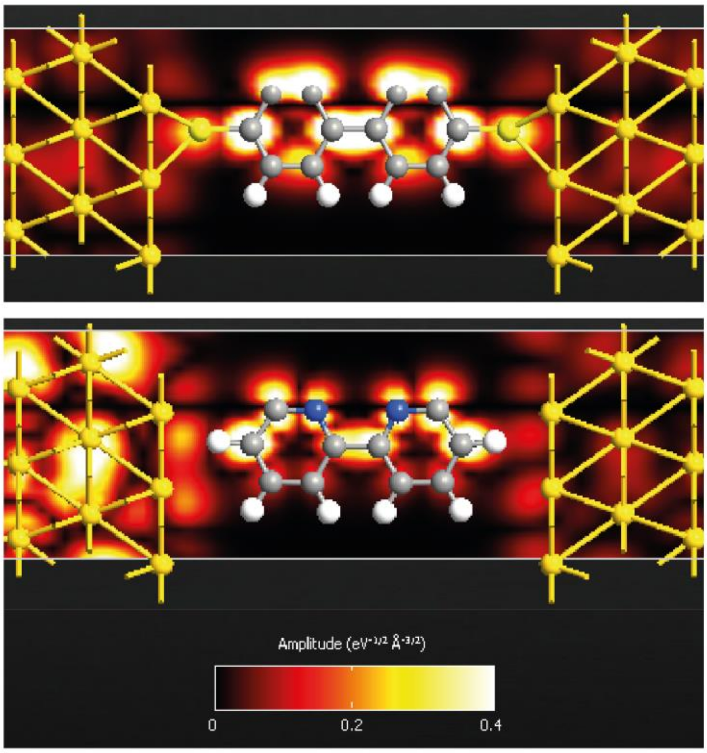

Supplementary Figure 7: Comparison between the transmission eigenstates for **I** and **IV**. **a,** Lowest transmission eigenstate for **I** at 0.88 eV. **b,** Lowest transmission eigenstate for **IV** at 4.8 eV. Calculated using *Virtual NanoLab* (VNL) and *Atomistix ToolKit* (ATK).

The coherent electron transport characteristics for 4 different MTJs were calculated using *Virtual NanoLab* with the *Atomistix ToolKit*.[3–6] The structures consist of two relaxed electrodes build from Au unit cells with a (111) surface. The final constructs (i.e. electrode-MTJ-electrode) were optimised with a DFT level theory (LDA) and electron transport properties were calculated for each of the MTJs using the extended Hückel basis set, resulting in the graphs shown in Figure 2c. Two examples of the lowest positive transmission eigenstates obtained from the electron transmission spectra, for **I** (at 0.88 eV) and **IV** (at 4.8 eV) are depicted in Supplementary Figure 7. The distance between the surface of the gold electrodes and the central molecular spacers changes with optimisation, which resulted in a slightly shorter distance between **IV** and the electrodes with respect to **I**, (Supplementary Figure 7). However, these calculations clearly represent a simplified version of our experiments, which in practice includes angled molecules and curved atomically-imperfect gold surfaces. Such variations will undoubtedly affect the absolute energy of each of the transmission eigenstates but will not differ enough to change the basic trends observed.

Supplementary Note 5: Width analysis

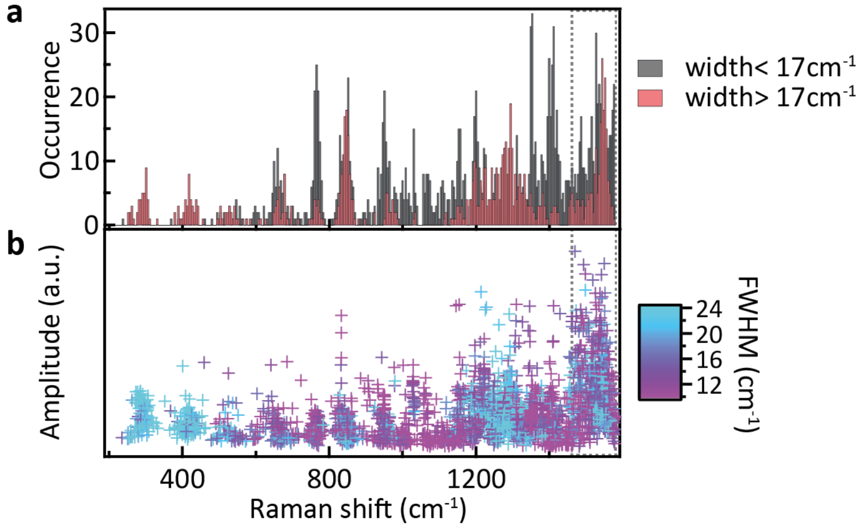

Supplementary Figure 8: SERS peakwidth analysis of the CB[8]-**VI** construct. Analysis obtained by fitting Gaussians to the data presented in Figure 4a**.** **a**, Histogram showing the occurrence frequency of peaks as a function of Raman shift, sorted into wide (> 17 cm^{-1}, red) and narrow (< 17 cm^{-1}, grey) widths. **b**, Amplitude of fitted peaks versus Raman shift, coloured according to their respective FWHM.

Supplementary Note 6: Chemical Reduction if VI

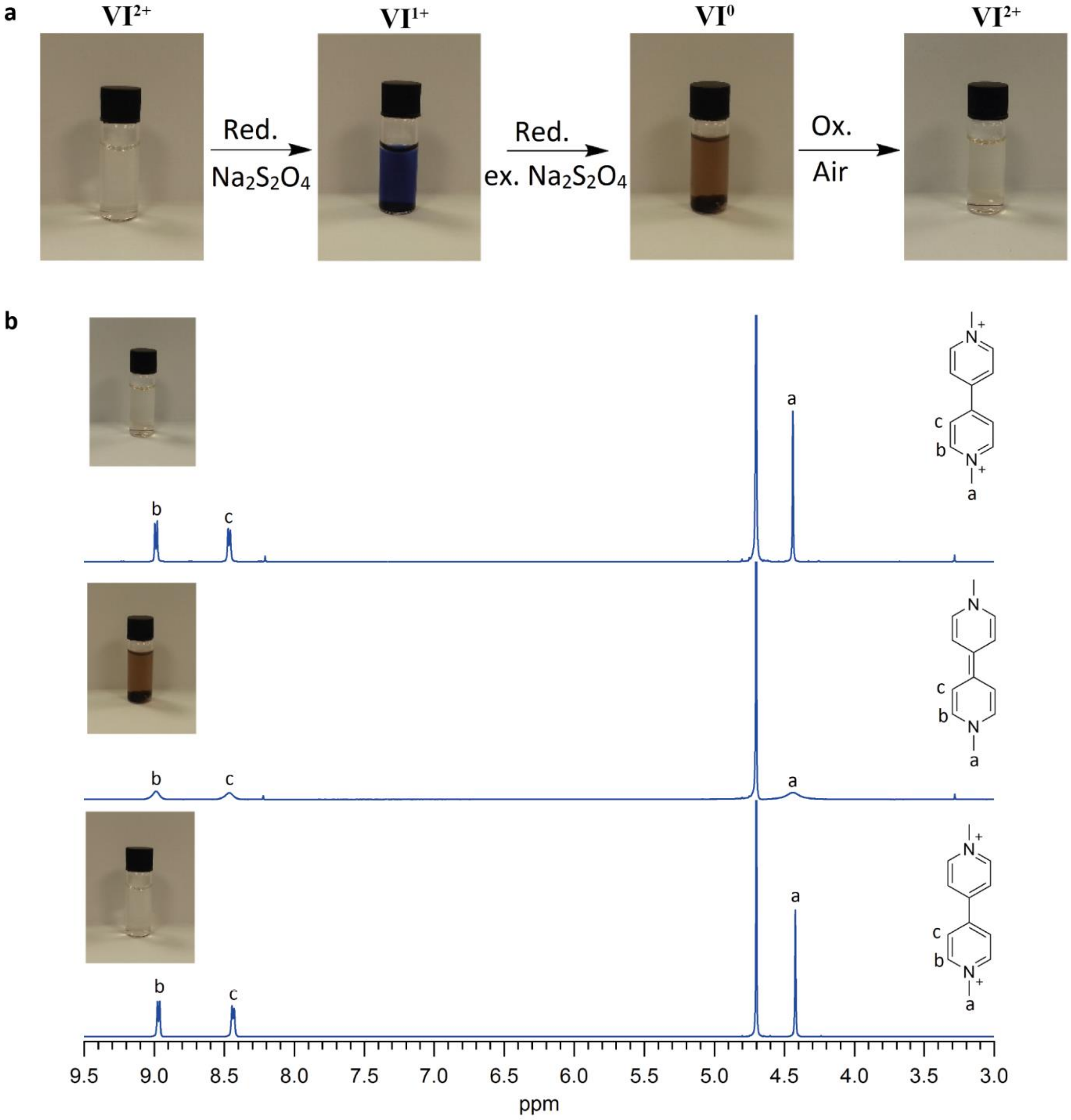

Supplementary Figure 9: NMR analysis of VI redox. **a,** Colour changes in a **VI** solution as sodium dithionite is added to reduce the $\mathbf{VI}^{2+}$ species to $\mathbf{VI}^{1+}$, then $\mathbf{VI}^{0}$. This is then oxidised back to $\mathbf{VI}^{2+}$ when open to air for 12 h. **b**, NMR spectra of $\mathbf{VI}^{2+}$ (lower spectrum), which is reduced to $\mathbf{VI}^{0}$ with sodium dithionite (middle spectrum), then left open to air for 12 h to oxidise back to $\mathbf{VI}^{2+}$ (upper spectrum). NMR spectra were collected using a 400 MHz Avance III HD NMR spectrometer.

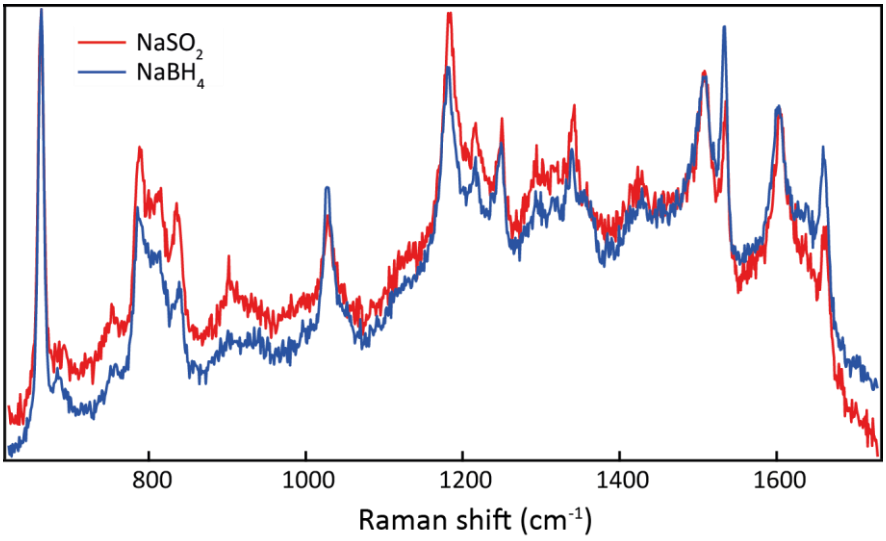

Supplementary Figure 10: **VI**^{0} SERS spectra resulting from chemical reduction of **VI**$^{2+}$. SERS spectra obtained for VI^0 using either $NaSO_2$ with CB[*n*] or $NaBH_4$ in the absence of CB[*n*] as reducing agent.

In the CB[*n*]-AuNPs aggregate system adding an excess of $NaSO_2$ gives a shift in the Raman spectrum upon illumination, from the 2+ to 0 redox state of **VI**. The same SERS spectrum is obtained when using $NaBH_4$ as the reducing agent (Supplementary Figure 10). Adding $NaSO_2$ to a AuNP solution containing **VI** but without CB[*n*] results in the formation of **VI**$^{1+}$. The conversion of **VI**$^{2+}$ to **VI**$^{1+}$ and **VI**^{0} whilst retaining the same molecular structure is confirmed using NMR (Supplementary Figure 9).

Supplementary References

1. de Nijs, B. *et al.* Unfolding the contents of sub-nm plasmonic gaps using normalising plasmon resonance spectroscopy. *Faraday Discuss.* **178,** 185–193 (2015).
2. Benz, F. *et al.* Single-molecule optomechanics in 'picocavities'. *Science* **354,** 726–729 (2016).
3. *Atomistix ToolKit, QuantumWise A/S, (www.quantumwise.com)*.
4. Brandbyge, M., Mozos, J.-L., Ordejón, P., Taylor, J. & Stokbro, K. Density-functional method for nonequilibrium electron transport. *Phys. Rev. B* **65,** 165401 (2002).
5. Soler, J. M. *et al.* The SIESTA method for ab initio order- N materials simulation. *J. Phys. Condens. Matter* **14,** 2745-2779 (2002).
6. Stokbro, K. *et al.* Semiempirical model for nanoscale device simulations. *Phys. Rev. B* **82,** 75420 (2010).
